# iCONE‐SRS: Development of inverse treatment planning for cone‐based stereotactic radiosurgery

**DOI:** 10.1002/acm2.12609

**Published:** 2019-05-16

**Authors:** Anthony Lausch, Brian Nghiem, Michelle Nielsen

**Affiliations:** ^1^ Medical Physics Department Carlo Fidani Peel Regional Cancer Centre Trillium Health Partners Mississauga Canada

**Keywords:** cones, inverse planning, simulated annealing, stereotactic radiosurgery

## Abstract

**Purpose:**

At present, commercially available treatment planning systems (TPS) only offer manual planning functionality for cone‐based stereotactic radiosurgery (SRS) leading to labor intensive treatment planning. Our objective was to reduce treatment planning time through development of a simple inverse TPS for cone‐based SRS.

**Methods:**

The iCONE TPS was developed using MATLAB (R2015a, The MathWorks Inc.) and serves as an inverse planning adjunct to a commercially available TPS. Simulated annealing is used to determine optimal table angle, gantry start and stop angles, and cone sizes for a user‐defined number of non‐coplanar arcs relative to user‐defined dose objectives. iCONE and clinically generated plans were compared through a retrospective planning study of 60 patients treated for 1–3 brain metastases (total of 100 lesions).

**Results:**

Planning target volume (PTV) coverage was enforced for all plans through normalization. PTV maximum dose was constrained to be within 120%–135% of the prescription dose. The median conformity index for iCONE plans was 1.35, 1.33, and 1.32 for 1, 2, and 3‐target cases respectively corresponding to a median increase of 0.05 (range = −0.1 to 0.5, *P* < 0.05), 0.06 (range = −0.83 to 0.53, *P* < 0.05), and 0.03 (range = −1.21 to 0.74, *P* > 0.05) relative to the clinical plans. No clinically significant differences were found with respect to the dose to organs‐at‐risk. Median iCONE planning times were approximately a factor of five lower than consensus estimates for manual planning provided by local experienced SRS planners.

**Conclusions:**

A simple inverse TPS for cone‐based SRS was developed. Plan quality was found to be similar to manually generated plans; however, degradation was observed in some cases highlighting the need for continued oversight and manual adjustment by experienced planners if implemented in the clinic. A factor of five reduction in treatment planning time was estimated.

## 1 INTRODUCTION

Stereotactic radiosurgery (SRS) has been shown to be an effective treatment for brain metastases^1^, ^2^ leading to an increase in demand and pressure on clinical resources. Effective treatment can be delivered using standard linear accelerators equipped with a set of small conical collimators;^3^, ^4^ however, both treatment planning and delivery can be labor intensive. Accordingly, there has been a push toward multi‐leaf collimator (MLC) based SRS which improves efficiency through the use of dedicated inverse planning tools and single isocenter deliveries (e.g. Varian HyperArc™, Brainlab Elements™ – Automatic Brain Metastases Planning).^5^, ^6^

Nonetheless, cone‐based SRS is still commonly employed and offers several attractive features such as a sharp penumbra, low transmission, and the reliability of a simple two‐part collimation system which does not move during treatment (e.g. simplified beam modeling, low delivery uncertainty, few device failure modes).

Unfortunately, unlike MLC‐based SRS, commercially available treatment planning systems (TPS) only offer forward planning functionality for cone‐based deliveries and so treatment planners must manually optimize several dozen parameters per treated target. This manual approach leads to prolonged treatment planning times particularly within the context of treating non‐spherical lesions. In addition, a significant learning curve is associated with becoming proficient with this type of planning. Meeks et al^7^ have previously reported a manual optimization algorithm to facilitate cone‐based SRS planning; however, their primary goal was to improve dose conformity for irregularly shaped lesions rather than to reduce treatment planning time.

Our objective was to address the challenge of prolonged treatment planning times for cone‐based SRS through the development of a simple inverse TPS which we refer to as iCONE. The iCONE algorithm is described and efficacy is demonstrated through retrospective application to data from patients previously treated for brain metastases.

## 2 METHODS

### 2.1 iCONE

#### 2.1.1 Workflow and interface

**Figure 1:**
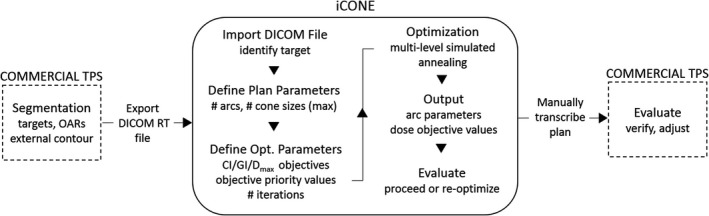
iCONE workflow.

**Figure 2:**
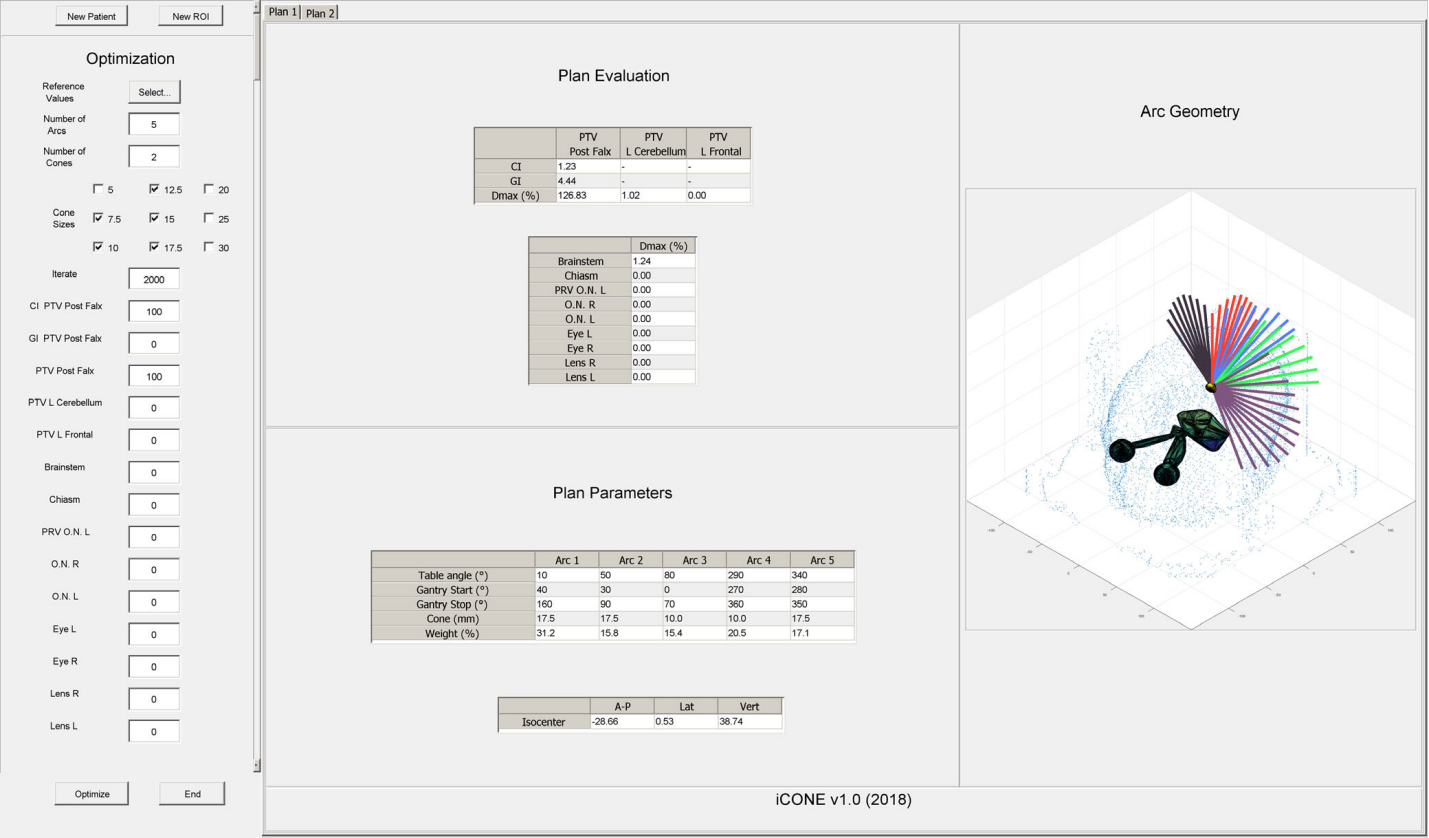
iCONE screenshot.

iCONE is a MATLAB‐based program (R2015a, The MathWorks Inc.) which serves as an inverse planning adjunct to a commercially available TPS. The iCONE workflow is summarized in Fig. 1 and a screenshot of the iCONE interface is shown in Fig. 2.

First, the user must delineate all targets, organs at risk (OARs), and an external or body contour in their respective commercial TPS and then export these structures in DICOM RT format. iCONE subsequently imports the DICOM RT file and the user identifies the target that they would like to generate a plan for (i.e. select planning target volume).

In iCONE, the user defines desired plan parameters including the number of non‐coplanar arcs and the maximum number of unique cone sizes to appear in the final plan (Fig. 2, “Optimization” panel). Default values were set to five arcs and a maximum of two unique cone sizes. iCONE automatically estimates the most relevant cone sizes to be considered for a given target; however, cone sizes can also be manually specified if desired. Relevant cone sizes are estimated by computing the diameter of a sphere with a volume equal to the target volume. The nearest‐diameter cone is identified and then the range of considered cone sizes is equal to this cone size plus or minus two cone sizes.

Next, the user defines optimization parameters which include the number of optimizer iterations and optimization objectives. For the selected target, the conformity index (CI), gradient index (GI), and maximum dose can be constrained.

CI is defined in iCONE as the ratio of the treated volume to the planning target volume (PTV) where the treated volume is equal to the total volume receiving the dose that must cover the PTV (D_cov_). The coverage dose D_cov_ has a default value of 95% of the prescribed dose but can also be specified by the user. Hereafter, CI_95_ will be used to denote the CI as computed using D_cov_ = 95%. The GI is defined as the ratio of the volume receiving 50% of the prescribed dose to the volume which receives 100% of the prescribed dose. The desired CI, GI, and maximum dose (D_max_) values can be specified along with optimization weights. For OARs, the maximum dose can be constrained to a specified value. The maximum dose to targets that are not being treated by the current plan can also be constrained in order to limit the interaction between plans generated for multi‐target cases.

iCONE then uses simulated annealing^8^ to search for a set of arc parameters which produces an optimal dose distribution with respect to the optimization objectives. During optimization, the CI, GI as well the maximum dose to all structures is displayed for the most optimal solution found at the time of the current iteration (Fig. 2, “Plan Evaluation” panel). Once optimization is complete, iCONE outputs the table angle, gantry start and stop angles, cone size, and weighting for each arc along with the isocenter coordinates (Fig. 2, “Plan Parameters” panel). A visualization of the arc geometry relative to the target and OAR structures is also provided (Fig. 2, “Arc Geometry” panel). The current plan can be re‐optimized using different objectives or alternatively a new plan can be created.

Creating a new plan “locks” the previous plan and generates a new plan tab which appears at the top of the window. Each locked plan can be reviewed for comparison purposes at any time by selecting its associated plan tab. Once a desirable plan has been found, arc parameters must then be manually transcribed into the commercial TPS by the user.

#### 2.1.2 Dose calculation

A simple correction‐based dose algorithm was implemented in iCONE. For simplicity and to reduce computation time, no heterogeneity correction was applied. Each arc is discretized into a series of beamlets for which the isocenter is defined as the PTV centroid. Beamlets are defined every 10°. The relative dose contribution of beamlet *b* at calculation point $\vec{x}$ is described by

$$D_{b}\left(\vec{x}\right)=\mathrm{TPR}\left(d\left(\vec{x}\right),c\right)\cdot \mathrm{RDF}\left(c\right)\cdot \mathrm{OAC}\left(\vec{x},c\right)\cdot {\left(\frac{\mathrm{SCD}}{\mathrm{SPD}(\vec{x})}\right)}^{2}$$

where $\mathrm{TPR}(d\left(\vec{x}\right),c)$ is the tissue phantom ratio evaluated at the calculation point depth $d(\vec{x})$ for cone *c*, RDF (*c*) is the relative dose factor for cone *c*, $\mathrm{OAC}(\vec{x},c)$ is the off‐axis correction evaluated for the point $\vec{x}$ and cone *c*, and ${\left(\frac{\mathrm{SCD}}{\mathrm{SPD}\left(\vec{x}\right)}\right)}^{2}$ is the inverse square law correction factor where SCD is the source‐to‐calibration distance and $\mathrm{SPD}\left(\vec{x}\right)$ is the source‐to‐calculation point distance for calculation point $\vec{x}$. TPR, RDF, and OAC beam parameters are derived from user‐tabulated data which has been previously measured in water. For simplicity, iCONE uses OAC data (i.e. profile data) acquired at a single depth for each cone and then scales the data according to beam divergence. Note that OAC is more commonly referred to as the “off‐axis ratio”; however, OAR denotes organ‐at‐risk in this study and so OAC has been used.

For a given arc and calculation point, the dosimetric contribution of all beamlets is computed and then summed together. The dose to the calculation point from all arcs is then summed. This process is repeated for all the calculation points in a 3D dose‐grid with 1 mm point spacing.

The dose to each grid point is subsequently normalized by the maximum dose value in the grid. This relative dose distribution (values range between 0 and 1) is then multiplied by D_cov_/D_99_ where D_99_ is the isodose line which covers 99% of the PTV. Recalling that D_cov_ is the required coverage dose for the PTV, the resulting distribution ranges between 0 and the maximum dose D_cov_/D_99_ with 99% of the target always covered by the dose D_cov_ (e.g. 99% of PTV covered by 95% of the prescription dose).

Note that an arc weighting parameter is not applied during arc summation. This corresponds to the assumption that an equal number of monitor units are delivered for every beamlet (i.e. dose‐rate is constant and equivalent for all arcs). iCONE‐reported arc weights are therefore derived rather than optimized parameters and are defined as the relative contribution of each arc to the dose at isocenter assuming a constant dose‐rate per unit arc length.

Two strategies were employed to improve dose calculation efficiency. First, the dose calculation grid was reduced to only include those points which fell within a 1 cm uniform expansion of the PTV. The points which define the OAR structures were then appended to the reduced grid. This modified grid permitted estimation of the CI, GI, and maximum dose to all structures while significantly reducing the number of calculation points. Second, a dose kernel approach was implemented whereby the dose to all grid points from all possible beamlets in the parameter space is computed prior to optimization. Dose calculation for a given arc geometry is then reduced to using a lookup table to sample the dose kernel and sum the dose contributions of each arcs’ beamlets.

#### 2.1.3 Optimization

The iCONE optimization parameter space corresponds to the set of all unique combinations of table angle, gantry start position, gantry stop position, and cone size for the user‐specified number of arcs. The range of considered table angles was restricted to [270°, 90°] to prevent collision with the gantry. For arcs with a table angle between 270° and 0°, the range of gantry angles was restricted to [0°, 180°] to reduce the risk of collision between the table and gantry. Conversely, gantry angles were restricted to [180°, 360°] for table angles between 0° and 90°.

The dose kernel is composed of beamlets defined at 10° table and gantry angle increments within this restricted space. The kernel contains pre‐computed beamlets at each of these angles for 5, 7.5, 12.5, 15, 17.5, 20, 25, and 30 mm diameter cones.

Simulated annealing coupled with a coarse‐to‐fine optimization scheme was used to search the parameter space for an optimal solution. The starting solution for each optimization was defined to be a set of equally spaced 180° non‐coplanar arcs each with the same cone size. This initial cone size was chosen to be the median size in the iCONE‐ or user‐specified range of cone sizes to be considered by the optimizer.

In the first or “coarse” level, a neighbor solution is generated by incrementing the table and gantry angle parameters of the current solution by 20°. In the second or “fine” level, angle parameters are incremented by 10°. The cone size increment is set to an increase or decrease in one size for both optimization levels. All plan parameters have an equal probability of changing or remaining the same when a neighbor solution is generated. The best plan found during the coarse optimization is then used to seed the fine optimization.

A weighted sum‐of‐squares objective function of the form

$$J=\sum_{i}w_{i}{\left(\frac{O_{i}-{\tilde{O}}_{\iota }}{{\tilde{O}}_{i}}\right)}^{2}$$

was used to inform the optimizer where ${\tilde{O}}_{\iota }$ is the objective for *i*‐th parameter and *O*_*i*_ is the value of that parameter for the plan considered in the current iteration. The {*w*_*i*_} are the user‐specified optimization priorities for each objective and are normalized by iCONE prior to optimization such that $\sum_{i}w_{i}=1$.

The acceptance probability for less optimal solutions was defined to be

$$P={\left(1+\exp\left[\Delta J/\left(c^{i}J_{o}\right)\right]\right)}^{-1}$$

where *∆J *= *J*_*i*_ – *J*_*i*−1_, *c* = 0.95, *i* is the iteration number, and *J*_0_ is the value of the objective function for the starting solution (*i *=* *0). This definition was selected to coincide with the MATLAB implementation of simulated annealing.^9^

Optimization was subject to several additional constraints. First, the minimum table angle separation between arcs was limited to 20°. Second, the minimum arc length was constrained to be 60°. Third, the sum of all arc lengths was constrained to be greater than or equal to 340°. These constraints were related to local planning procedures and so were hardcoded in iCONE; however, they can be changed to assume any value.

### 2.2 Evaluation

#### 2.2.1 Dataset

Treatment planning data from 60 patients previously treated with cone‐based SRS for brain metastases were used for this retrospective study. Thirty cases were treated for a single metastasis, 20 cases were treated for two metastases, and 10 cases were treated for three metastases for a total of 100 treated targets. Cases were selected at random with a preference for more recently treated patients since the local SRS program was initiated in 2014 and planning expertise was assumed to have increased over time. Table 1 summarizes the location of the 100 lesions.

**Table 1:** Location of the 100 investigated lesions

Location	#
Frontal	42
Cerebellar	16
Temporal	12
Occipital	11
Parietal	9
Brainstem	2
Misc	8

Organs at risk and gross tumor volumes (GTVs) were previously delineated by experienced treatment planners and radiation oncologists respectively. A 2 mm PTV margin was used to account for known setup uncertainty. The median PTV volume was 1.6 cc (range = 0.3–10.9 cc). The median prescription dose was 15 Gy (range = 11–21 Gy) with lower doses associated with OAR‐target proximity, larger target volumes, and multi‐target cases.

#### 2.2.2 Treatment planning

The clinically delivered treatment plans were originally generated using the iPlan TPS (version 4.5.6, Brainlab) which employed a correction‐based dose model with heterogeneity correction. The planning constraints that were used during generation of the clinical plans are summarized in Table 2 and represent the minimum requirements for an acceptable plan. Brain dose constraints are not listed in Table 2 since a tumor size‐based triage is typically applied at our centre whereby larger tumors are treated with fractionated stereotactic radiotherapy rather than SRS. However, for completeness, we report V12 values (volume of the healthy brain receiving 12 Gy) for both clinical and iCONE‐generated plans within this study.^10^

**Table 2:** Treatment planning constraints

Structure	Constraint
PTV	D99% = 95%
	120% < D_max_ < 135%
	1 < CI_95_ < 2
	GI < 4
Brainstem	D_max_ < 12 Gy
Lens	D_max_ < 2 Gy
Optic nerves	D_max_ < 8 Gy
Optic chiasm	D_max_ < 8 Gy
Eyes	D_max_ < 8 Gy

D_max_ denotes maximum point dose, CI_95_ denotes conformity index computed using the volume receiving 95% of the prescription dose, and GI denotes the gradient index.

In addition to dose constraints, local clinical protocol also required a minimum table angle separation of 20° between arcs and that the sum of all arc lengths be at least 340°. Finally, the maximum number of monitor units (MUs) to be delivered from a single table angle was limited to 999.

The ROI structure files for the N = 60 patients were exported from Brainlab iPlan in DICOM RT format. iCONE was subsequently used to generate plans for each PTV based on the structure files. The planner was blind to the clinical plan quality during iCONE plan generation and attempted to minimize dose to OARs as much as possible while maintaining acceptable target CI_95_ and D_max_ values. PTV D99% = 95%, table angle separation, and arc length constraints are necessarily met by all iCONE plans as these constraints were hard‐coded. The total number of optimizer iterations was set to a constant value of 4000 (2000/optimizer level by two levels) for all optimizations.

#### 2.2.3 Analyses

The primary analyses made were with respect to plan quality, plan complexity, and treatment planning time. For plan quality, the Table 2 dose parameters are reported and compared for the iCONE and clinical plans. Dose parameters were evaluated within the commercial TPS for both iCONE and clinical plans. This required transcription of iCONE plans into Brainlab iPlan and subsequent recalculation of the delivered dose using the iPlan dose engine. Plan complexity was evaluated by comparing the number of arcs and unique cone sizes utilized by different plans. A complexity score (CS) was assigned to each plan which was equal to the sum of the number of unique cone sizes and the number of arcs used by the plan (i.e. CS = N_arcs_ + N_cones_).

Treatment planning time was measured for iCONE‐generated plans and then compared to a consensus estimate for manual treatment planning time provided by local treatment planners.

The consensus estimate was defined as the total time required to manually optimize plan parameters whereas the planning time for iCONE was defined as the total time required to export the ROI DICOM RT file, optimize plan parameters, and then manually transcribe the treatment plan back into the commercial TPS. The Wilcoxon signed‐rank test was used to assess statistical significance for all comparisons.

## 3 RESULTS

### 3.1 Plan quality

**Figure 3:**
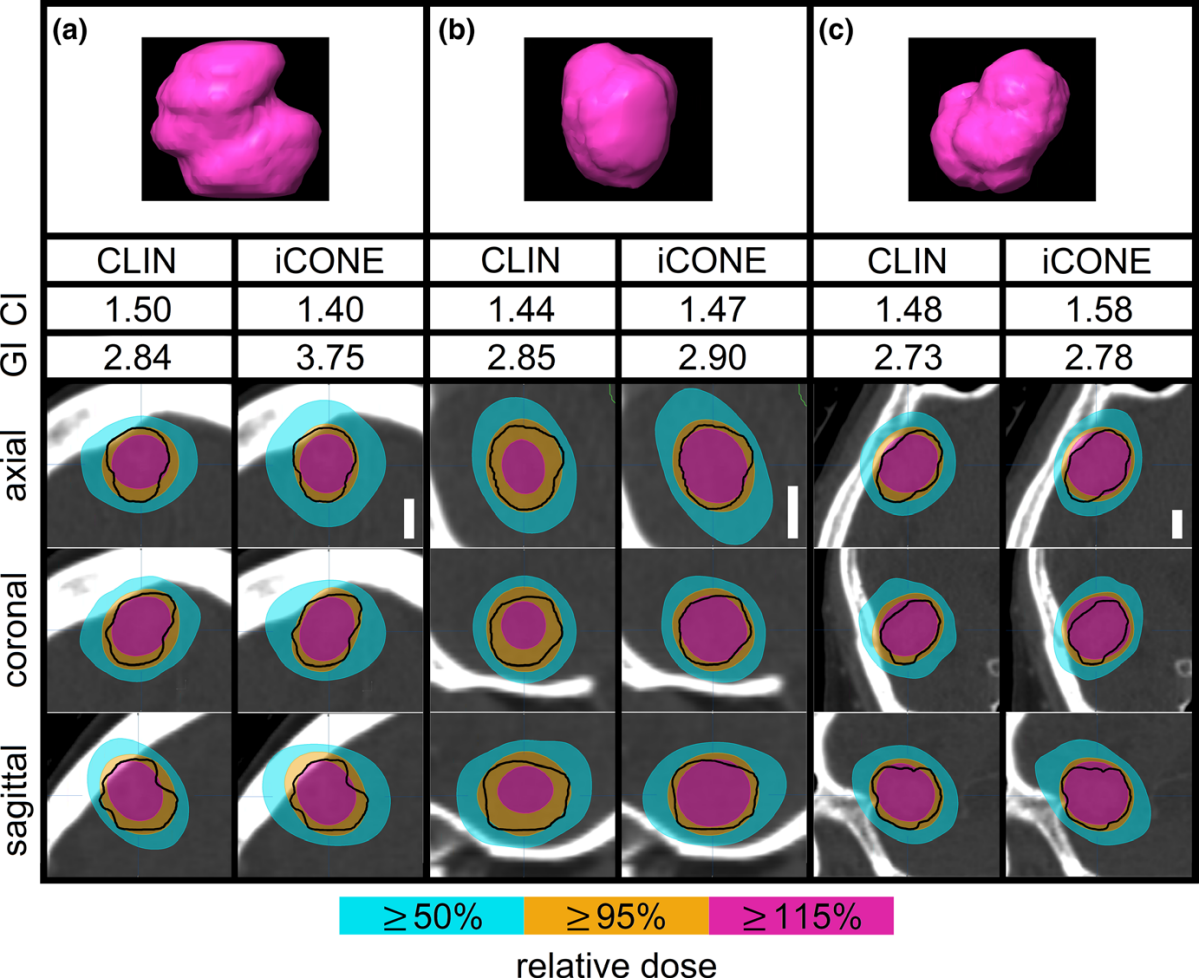
Relative dose distributions from clinical (CLIN) and iCONE plans generated for (a) a frontal lobe lesion, volume = 2.9 cc, (b) cerebellar lesion, volume = 1.7 cc, and (c) another frontal lobe lesion, volume = 6.0 cc. The planning target volume is delineated by a solid black line. The white scale bars represent 1 cm and each bar is applicable to all of the images for a given lesion. CI and GI refer to conformity and gradient index respectively.

Stereotactic radiosurgery plans were generated using iCONE for the N = 60 cases (100 targets) and compared to the clinically delivered plans (CLIN). Relative dose distributions are shown for three different lesions in Fig. 3. Examples are provided where iCONE decreased the CI (i.e. improved conformity, Fig. 3a), maintained similar CI (Fig. 3b), and increased the CI (i.e. reduced conformity, Fig. 3c) relative to the CLIN plans. These cases illustrate challenging lesions to plan as evidenced by their irregular shape and the resulting CIs which were in the upper‐quartile of all values. The axial, sagittal, and coronal planes sampled for each distribution were positioned close to the centre of the lesions.

CI_95_ constraints were unmet (CI_95_ > 2) for 2/100 and 1/100 lesions for CLIN and iCONE‐generated plans respectively. For CLIN‐generated plans, PTV‐maximum dose constraints were unmet for 30/100 lesions where the maximum dose was found to be lower than 120% for all 30 lesions. Twenty‐four out of 30 lesions where a lower maximum dose was observed occurred within the context of multi‐target treatment. For iCONE‐generated plans, PTV maximum dose constraints were unmet for 10/100 lesions where the maximum dose was lower than 120% for 2/10 lesions and exceeded 135% for 8/10 lesions. For 7/8 lesions, the PTV maximum dose constraint was exceeded within the context of multi‐target treatment. The gradient index constraint (GI < 4) was unmet for 1/100 and 7/100 lesions for CLIN and iCONE‐generated plans respectively with all iCONE instances corresponding to multi‐target treatment.

**Figure 4:**
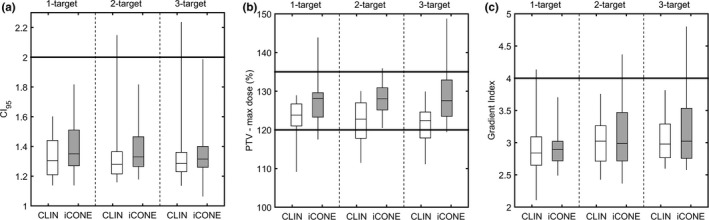
Distribution of (a) CI_95_, conformity index computed using volume receiving 95% of the prescription dose, (b) PTV maximum point dose, and (c) gradient index values for 1, 2, and 3‐target cases. Note that PTV coverage was enforced for all cases through renormalization. Error bars span the central 95% of each distribution. The thick horizontal lines indicate constraints from Table 2.

Figure 4 indicates the distribution of CI_95_, maximum PTV dose, and gradient index values for CLIN and iCONE generated plans. For multi‐target cases, the maximum dose to the PTV was evaluated based on the sum of the dose from all plans. For CLIN plans, the median CI values were 1.31, 1.28, and 1.29 for 1, 2, and 3‐target cases respectively. For iCONE plans, the median CI values were 1.35, 1.33, and 1.32 for 1, 2, and 3‐target cases respectively. The median CLIN PTV‐maximum dose values were 123.8%, 122.8%, and 122.4% while the median iCONE PTV‐maximum dose values were 128.2%, 128.1%, 127.6 for 1, 2, and 3‐target cases respectively. The median CLIN gradient index values were 2.8, 3.0, and 3.0 while the median iCONE gradient index values were 2.9, 3.0, and 3.0 for 1, 2, and 3‐target cases respectively.

The differences between iCONE and CLIN values from Fig. 4 were computed and are displayed in Table 3. A small increase in CI_95_ was observed for iCONE generated plans which was found to be statistically significant for 1‐ and 2‐target cases (*P* < 0.05) but not for 3‐target cases. A statistically significant increase in the PTV maximum dose was observed for 1, 2, and 3‐target cases planned using iCONE while no significant differences were observed with respect to the gradient index.

**Table 3:** Median difference between iCONE and Clinical CI_95_, PTV maximum dose (D_max_), and gradient index (GI) values for 1, 2, and 3‐target cases

# targets	ΔCI_95_	ΔD_max_	ΔGI
1	0.05 [−0.10, 0.50]^a^	3 [−6, 21]^a^	0.02 [−1.53, 0.97]
2	0.06 [−0.83, 0.56]^a^	5 [−7, 20]^a^	0.02 [−1.07, 1.51]
3	0.03 [−1.21, 0.74]	7 [−3, 28]^a^	0.05 [−0.85, 2.20]

Statistically significant differences are indicated and the range of differences is reported in brackets. For multi‐target cases, D_max_ values were computed based on the sum of the dose from all plans.

a Indicates *P* < 0.05.

**Figure 5:**
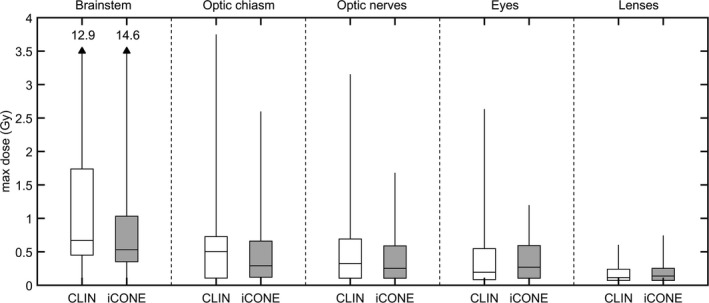
Maximum point doses for organs at risk. Values were computed for multi‐target cases based on the sum of the dose from all plans. Error bars span the central 95% of each distribution except for the brainstem plots where the upper bound is indicated numerically to facilitate visualization of the other distributions.

Figure 5 indicates the distribution of OAR maximum doses for CLIN and iCONE generated plans. Values were computed for multi‐target cases based on the sum of the dose from all plans. The brainstem constraint was unmet (max dose > 12 Gy) for 2/60 and 3/60 cases for CLIN and iCONE‐generated plans respectively. These exceptions occurred within the context of brainstem or brainstem‐abutting tumor treatment and both cases observed for CLIN were also observed for iCONE. The optic nerve constraint was exceeded in 1/60 cases for both CLIN and iCONE‐generated plans due to the number of treated lesions (N = 3) in combination with lesion proximity to the left optic nerve.

Table 4 summarizes the differences between the distributions in Fig. 5. A small but statistically significant increase in the maximum lens dose (median = +0.01 Gy) was observed in iCONE plans. Maximum dose was not found to be different between CLIN and iCONE‐generated plans for any of the other OAR structures constrained in Table 2.

**Table 4:** Median difference between iCONE and clinical maximum doses (D_max_) for organs at risk

	ΔD_max_ (Gy)
Brainstem	−0.09 [−3.1, 2.3]
Optic chiasm	0.00 [−1.2, 1.0]
Optic nerves	0.00 [−3.2, 1.4]
Eyes	0.01 [−2.9, 0.9]
Lenses	0.01 [−0.7, 0.6]^a^

Statistically significant differences are indicated and the range of differences is reported in brackets.

a Indicates *P* < 0.05.

For the healthy brain, the median V12 values for clinical and iCONE plans were 4.7 cc (range = 0–17.9 cc) and 4.5 cc (range = 0–21.3 cc) respectively. The median difference was −0.2 cc (range = −7.5 to 6.8 cc) which corresponded to a small but statistically significant decrease in the iCONE plans (*P* < 0.05).

### 3.2 Plan complexity

The median number of arcs used per treated lesion was 5 (range = 5–12) and 6 (range = 5–7) for CLIN and iCONE‐generated plans respectively. The median number of unique cone sizes used per treated lesion was 2 (range = 1–3) and 2 (range = 1–2) for CLIN and iCONE‐generated plans respectively. The median plan CS was 7 (range = 6–14) and 8 (range = 6–9) for CLIN and iCONE‐generated plans respectively with a median difference of + 1 (range = −7 to 3) in the iCONE plans.

### 3.3 Planning time

iCONE treatment planning was performed using an Intel Core i7‐6700 desktop computer operating at 3.4 GHz with 4 GB RAM and 64‐bit Windows. For a standard 5‐arc, 2‐cone size plan, 4000 optimizer iterations required approximately 80–100 s of computation time with longer times corresponding to larger volume targets. Multiple optimizer runs were performed using different optimization priorities to arrive at a final plan for each target similar to inverse‐planning for MLC‐based deliveries. ROI DICOM RT file export and plan transcription steps were observed to require one additional minute per patient and approximately two additional minutes per treated lesion respectively. Table 5 indicates the median treatment planning time for iCONE‐generated plans alongside a consensus estimate provided by local SRS planners for the optimization time required by the conventional manual approach. The tabulated iCONE values are equal to the sum of the measured iCONE optimization times and the aforementioned ROI DICOM RT file export and plan transcription time estimates.

**Table 5:** Median treatment planning time for iCONE plans including ROI export, plan optimization, and plan transcription steps

# targets	iCONE (min)	CLIN (min)
1	8 [5, 30]	45
2	19 [10, 41]	90
3	25 [17, 51]	135

Ranges are reported in brackets. Clinical values are a consensus estimate provided by local SRS treatment planners for the time required by the conventional manual optimization approach.

## 4 DISCUSSION

A simple inverse‐treatment planning system called iCONE was developed to address the challenge of prolonged treatment planning times for cone‐based SRS. To our knowledge, this is the first study to report the application of inverse optimization techniques to cone‐based SRS planning. The new tool was applied retrospectively to data from 60 patients previously treated for 1–3 brain metastases. Performance was assessed with respect to plan quality, complexity, and planning time relative to the clinically delivered treatments.

iCONE plan quality was found to be comparable to clinically generated plans with the exception of a median increase in CI_95_ of between 0.03 and 0.06 depending on the number of treated targets (Table 3). PTV maximum dose values were higher than clinical plans; however, the distribution of values was better centered within the clinically desirable range (Fig. 4b). No statistically significant differences in GI were observed for iCONE plans suggesting similar dose fall‐off characteristics. This is further corroborated by the healthy brain V12 values which were found to be nearly equivalent to clinical values (median decrease in 0.2 cc in iCONE plans). iCONE and clinical OAR maximum doses were similar with the exception of a small but statistically significant median increase in lens dose of 0.01 Gy. It is noteworthy that the user who generated all iCONE plans had no previous experience with radiotherapy treatment planning.

Overall, iCONE met planning constraints with a similar frequency compared to clinically generated plans. The 100 lesions in this paper were selected at random and so included both OAR‐proximal and OAR‐distal cases. For proximal cases, OAR planning objectives were frequently necessary to reduce OAR dose. For distal cases, OAR objectives were not strictly necessary to meet constraints but were still employed to keep dose as low as reasonably achievable (ALARA). Demonstrating that ALARA principles can be preserved is of particular importance for cranial SRS since patients may undergo treatment for additional lesions leading to larger aggregate doses despite lower doses in individual plans.

While iCONE plan quality was similar to clinical plans, plan complexity was found to be higher. Increased plan complexity was typically due the addition of an extra arc. The number of arcs and unique cone sizes used by the optimizer is defined by the user a‐priori so that they can directly control plan complexity. Within this study, an extra arc was primarily added to ensure that no more than 999 monitor units were delivered at a single couch angle due to a local planning constraint rather than to further improve dose metrics. An alternative approach could have involved using fewer arcs followed by incrementally decreasing the weight of the offending arc once the plan was transferred into the commercial TPS.

The primary objective of this study was to reduce cone‐based SRS treatment planning times through development of inverse planning. Median iCONE planning times were found to be approximately a factor of five lower than consensus estimates for manual planning (Table 5) suggesting potential for significant planning time reduction. Furthermore, the number of optimizer iterations used in this study (N = 4000) was not determined a‐priori to offer the best trade‐off between optimization time and solution quality. Subsequent testing has suggested that similar quality plans could be generated using half as many iterations which could lead to a further factor of two reduction in iCONE planning time.

There are several important limitations to consider when interpreting the results of this study. Principal among these is the potential for discrepancies between iCONE and commercial TPS derived estimates of plan quality metrics. In practice, the user will rely on iCONE‐estimated metrics to decide when they have arrived at a suitable solution and should transcribe the plan to a commercial TPS. However, due to differences between the geometry representation and dose calculation methods employed by iCONE and the commercial TPS, the value of these metrics can change upon final evaluation in the commercial TPS. For example, Brainlab iPlan employs an adaptive resolution scheme that uses a finer dose grid near steep gradients whereas iCONE uses a uniform grid in order to enable the use of pre‐computed dose kernels and rapid dose calculation.

In view of this limitation, we only report commercial TPS‐derived quality metrics for iCONE plans and also did not permit re‐optimization in the event that reduced quality was observed in the commercial TPS. All results reported in this study include any potential changes in plan quality upon transcription so that the reader can properly assess the current clinical utility of iCONE. This effect is manifest primarily in the reported CI and maximum target dose values. For example, the median CI_95_ over all 100 lesions as initially estimated by iCONE was 1.27 (IQR = 1.20–1.39) which was not statistically different from the Brainlab iPlan‐estimated CI_95_ values for the clinical plans (*P* > 0.05, median = −0.01, IQR = −0.08 to + 0.06). However, upon re‐evaluation of iCONE plans in Brainlab iPlan there was a statistically significant increase in CI_95_ values (*P* ≪ 0.01, median = +0.05, IQR = 0–0.10). Similarly for the maximum dose to the target, the iCONE estimated median was 125% (IQR = 123%–127%) but there was a statistically significant increase upon re‐evaluation in Brainlab iPlan (*P* ≪ 0.01, median = +0.1%, IQR = 0–4%).

This result has several implications. First and foremost, the possibility for quality degradation underscores the importance of the evaluation step in the Fig. 1 workflow. Continued oversight by experienced planners is essential to ensure that the plan in the commercial TPS realizes the plan quality as estimated by iCONE. This may necessitate manual adjustment and increase planning time. Second, iCONE‐estimated plan quality appears to be equal to the plan quality attainable via current manual methods and so iCONE estimates can provide guidance on the quality that is possible for a given case. Third, increased knowledge and incorporation of commercial TPS methodologies or direct integration into a commercial TPS could improve utility via increased concordance in quality metric estimation.

Other current iCONE limitations include no split‐arc support, no isocenter position or beam weighting optimization, serial optimization of multiple targets, and no computation of absolute dose. Despite these limitations, favorable results were still found in this study and so supervised iCONE‐use will begin to be investigated in our local workflows with functionality to be expanded in future versions if desired by the clinic.

## 5 CONCLUSION

A simple inverse treatment planning system for cone‐based SRS called iCONE was developed. Plan quality was found to be similar to manually generated plans in a retrospective analysis of patients treated for 1–3 brain metastases, however, quality degradation was observed in some cases highlighting the need for continued oversight and manual adjustment by experienced planners. A factor of five reduction in treatment planning time was estimated if iCONE were to be integrated into the local clinical workflow.

## CONFLICT OF INTEREST

No conflicts of interest.
